# The implementation of the free maternal health policy in rural Northern Ghana: synthesised results and lessons learnt

**DOI:** 10.1186/s13104-018-3452-0

**Published:** 2018-05-29

**Authors:** Philip Ayizem Dalinjong, Alex Y. Wang, Caroline S. E. Homer

**Affiliations:** Faculty of Health, University of Technology Sydney, Ultimo, Sydney, NSW Australia

**Keywords:** National Health Insurance, Free maternal health policy, Fee exemption, Maternal health services, Pregnancy, Childbirth, Lessons, Ghana

## Abstract

**Objective:**

A free maternal health policy was implemented under Ghana’s National Health Insurance Scheme to promote the use of maternal health services. Under the policy, women are entitled to free services throughout pregnancy and at childbirth. A mixed methods study involving women, providers and insurance managers was carried out in the Kassena-Nankana municipality of Ghana. It explored the affordability, availability, acceptability and quality of services. In this manuscript, we present synthesised results categorised as facilitators and barriers to access as well as lessons learnt (implications).

**Results:**

Reasonable waiting times, cleanliness of facilities as well as good interpersonal relationships with providers were the facilitators to access. Barriers included out of pocket payments, lack of, or inadequate supply of drugs and commodities, equipment, water, electricity and emergency transport. Four lessons (implications) were identified. Firstly, out of pocket payments persisted. Secondly, the health system was not strengthened before implementing the free maternal health policy. Thirdly, lower level facilities were poorly resourced. Finally, the lack of essential inputs and infrastructure affected quality of care and therefore, access to care. It is suggested that the Government of Ghana, the Health Insurance Scheme and other stakeholders improve the provision of resources to facilities.

## Introduction

A free maternal health policy was implemented in Ghana in July 2008 under the National Health Insurance Scheme (NHIS). The policy allows all pregnant women to have free registration with the NHIS after which they would be entitled to free services throughout pregnancy, childbirth and 3 months postpartum. The policy was one of Ghana’s key strategies for the achievement of the Millennium Development Goals (MDGs) and now, the Sustainable Development Goals (SDGs), specifically the reduction of maternal and child deaths and the achievement of universal health coverage (UHC).

It is unclear whether the policy has achieved its desired outcomes in all parts of Ghana. In other resource constrained settings, it has been shown that there are gaps in similar policy implementation, as these are often implemented without careful planning and inadequate infrastructure as well as resources in terms of workforce and funding [1–3]. Implementation is often affected by factors inside and outside the health system, which ultimately affects access to services.

Access to services is complex and multidimensional [4] and is determined by factors in the health system as well as at the individual, household and community level [5, 6]. The dimensions of access are classified broadly as affordability, availability, acceptability and quality of care. These affect the use and provision of services and are key for the successful implementation of policies. Therefore, we undertook a study to explore the affordability, availability, acceptability and quality of services under the free maternal health policy. Some of the results have been published in [7, 8], specifically those relating to affordability. In this manuscript, we present the overall synthesised results in the form of facilitators and barriers to access to services under the free maternal health policy. In addition, we highlight the key lessons (implications) drawn from the study.

## Main text

### Methods

The study was cross-sectional, combining quantitative and qualitative studies using the convergent parallel mixed methods design. The study area was the Kassena-Nankana municipality in rural Northern Ghana. Quantitative data were collected from women (n = 406) who gave birth in facilities and at home. In-depth interviews were conducted among providers and insurance managers (n = 28), while focus group discussions were held with the same category of women (n = 10) who participated in the quantitative study. Details of the design, study area, sampling, data collection and analysis are published in [7, 8].

### Results

**Fig. 1:**
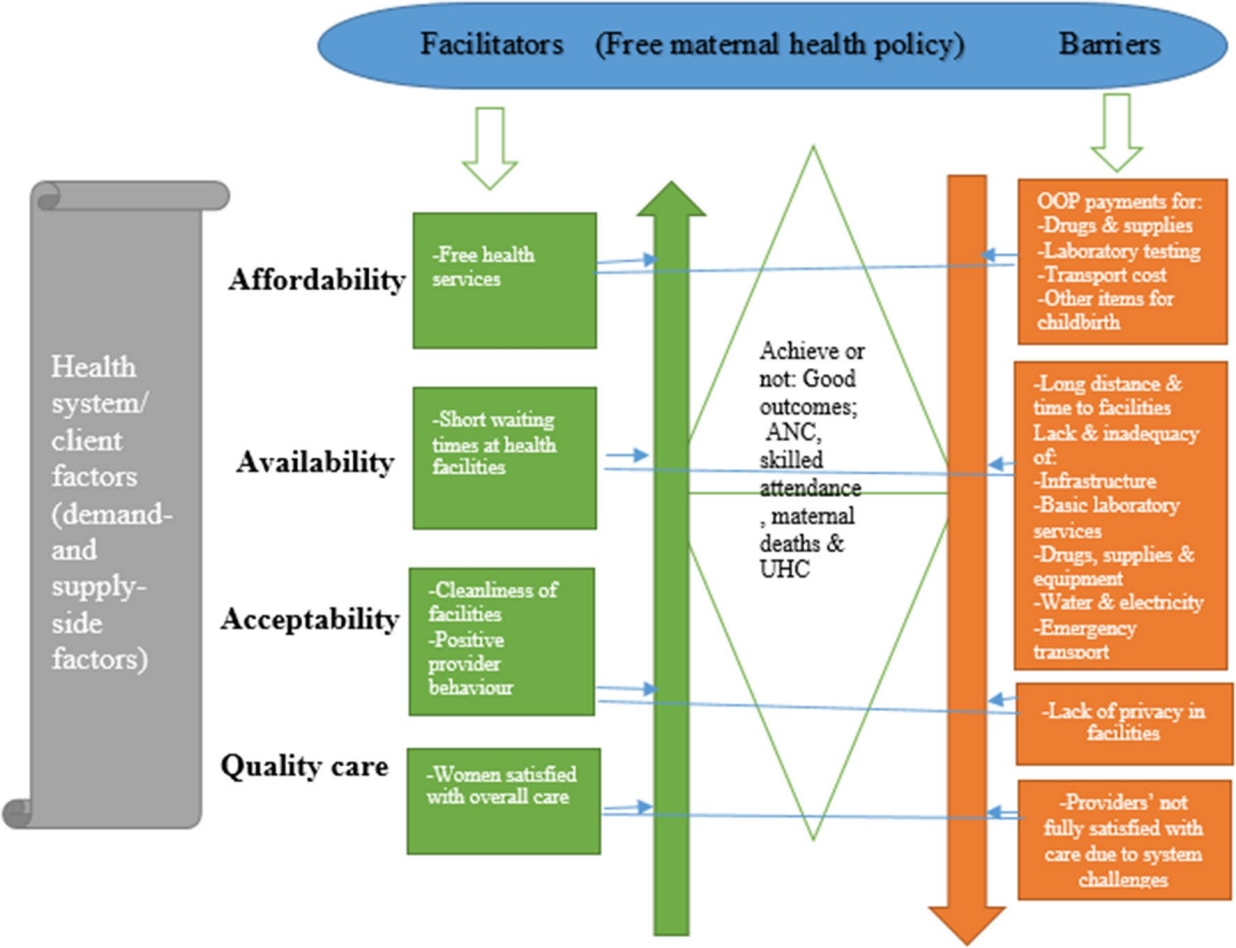
Synthesis of study results

The results have been synthesised and categorised as facilitators and barriers to access in terms of; affordability, availability, acceptability and quality of care. Figure 1 represents the overall synthesised results.

### Discussion

#### Facilitators of access to services under the policy

The benefits of the free maternal health policy was widely acknowledged as it promoted the use of services. Other reviews in low- and middle-income settings have reported a significant positive relationship between health insurance including fee exemptions and the use of services [9, 10]. Interestingly, in our study waiting times were perceived not to impede the use of services. This is contrary to results from studies in Kenya [11], India [12] and Lao People’s Democratic Republic [13]. The result may have been because women in this area expected to wait and had very few or limited expectations about what the service would provide.

Equally, we found that the facilities were reported to be clean and providers to be respectful and friendly. The environment of facilities as well as the attitudes of providers are important predictors of service usage. A review on the determinants of women’s satisfaction with services in low- and middle-income countries has shown that the interpersonal relationships of providers dominated factors influencing women’s use of services [14]. It is encouraging that women in this rural region were positive about their relationships with providers but this may again be attributed to low expectations.

Women interviewed indicated that they were very satisfied or satisfied with quality of care. This result is in line with studies conducted in India and Bangladesh where women were reportedly satisfied with services provided under the Chiranjeevi and the Maternal Health Voucher Schemes respectively [15, 16]. However, the result runs contrary to other studies, for example from Bangladesh where women expressed dissatisfaction with quality of services received [17]. Satisfaction is a difficult concept and is dependent on expectations and the outcome [18]. For example, many women report being ‘satisfied’ at the time of care merely because they and their babies survived the experience and sometime later, they articulate a more nuanced, usually negative, experience. This is the halo effect of maternity care [19–21] and may be responsible for our positive results. Despite the high rates of satisfaction, many women in our study also reported a lack of privacy during labour and birth suggesting that perhaps their ‘satisfaction’ was actually limited.

#### Barriers for access to services under the policy

Our study demonstrated that, despite the policy, women still made out of pocket (OOP) payments for drugs, supplies, laboratory services including ultrasound scans and transport as well as the purchase of other items for childbirth. The results corroborate findings from similar settings. For instance, despite a policy in Ethiopia to provide free services for women, 65% of facilities required women to make payments for some services [22] and in Senegal, where women made payments for transport and drugs under the Free Delivery and Caesarean Policy [23]. These highlight the challenges with implementing fee exemption policies in many countries.

Distance and time taken to reach the nearest facility were perceived in our study to be impediments to care seeking. The result is not isolated. In South Africa and Zambia, women revealed long distances to facilities which hindered access [24, 25]. Likewise, basic essential inputs such as infrastructure, laboratory tests, drugs and supplies, equipment, water, electricity and emergency transport were either inadequate or unavailable in many of the lower level facilities; that is, the community-based health planning and services (CHPS compounds).

While women reported being satisfied with care, this was not the case for the providers. Providers recognised that the situation meant that the care they were providing was sub-standard. Providers often know what ‘good’ care should be even if their clients are willing to accept less than ‘good’. Other studies have highlighted similar issues, for example, in Bangladesh; while women reported satisfaction with services, providers were unhappy with care provision due to staff and logistics challenges, the lack of laboratory services and insufficient supervision [26].

#### Lessons learnt (implications) from the results

Although the Government of Ghana has prioritised maternal health by implementing the policy a decade ago, the results of our study raise critical questions about the ability of the policy to meet its goals. Our study highlighted four useful lessons for policy makers and other stakeholders in Ghana. These lessons are relevant to other countries who have implemented or are planning to implement fee free policies.

#### Lesson number one: OOP payments persisted despite the NHIS

OOP payments were common. The cost of transport, laboratory services, drugs and supplies made service utilisation difficult especially for poor women. The lack of funds in facilities was a result of the delay in payments by the NHIS, partly caused by the claims process and to some extent the lack of adequate funds for the scheme. The establishment of the electronic claims submission system by the NHIS is a step in the right direction as this will reduce fraud and abuse, help contain costs and promote the financial sustainability of the NHIS [27, 28]. The system also allows for the early settlement of claims, thereby encouraging them to continue to provide services to clients of the NHIS.

Sustainable sources of funding to ensure funds are available for claims payment within the stipulated time (1 month following submission to the NHIS). Currently, the NHIS relies on a 2.5% value added tax (Health Insurance Levy) on some categories of goods and services as one of its main sources of funding [29]. An additional 1% increase in the levy is suggested to raise more money for the smooth operation of the NHIS. The greatest need is to ensure efficiency, as more funding does not necessarily imply the success of the NHIS. Measures should be put in place to identify poor women as a priority for the reimbursement of the cost of transport to facilities, although the process of prioritisation for reimbursement will need attention. Reimbursing the transport cost for women who are poor, in addition to the benefit package of the policy, may encourage their use of services.

#### Lesson number two: a weak health system challenged access

The inadequacy or unavailability of drugs and supplies, equipment, transport and infrastructure meant the health system was unable to support the successful implementation of the policy. This is synonymous with settings in low- and middle-income countries, where the outbreak of epidemics and other emergencies, for example, the outbreak of the Ebola Virus in West Africa, exposed the vulnerability and weaknesses of the health system [30, 31]. Strong health systems are required to attain health goals [32, 33], provide routine or usual services and to contain disease outbreaks [34, 35]. Such strong health systems provide the assurance that the required workforce, equipment, drugs and supplies, transport, information, monitoring and supervision, affordable and responsive services as well as good provider relations exist in the process of service delivery [36]. The success of Ghana’s policy requires an ongoing investment in drugs and supplies, equipment and transport as well as improvement in the infrastructure of facilities.

#### Lesson number three: lower level facilities are poorly resourced

Lower level facilities (CHPS compounds) in the study are poorly resourced for the provision of services to people living in distant and remote communities. Nevertheless, these play a crucial role, acting as gatekeepers to the health system and as the first point of care for women, including the poor. These facilities also provide basic preventive and curative services. Thus, strengthening peripheral health systems is key to the achievement of good health outcomes as well as the attainment of UHC. Our study highlights the need for an expansion in the infrastructure of the CHPS compounds, including the provision of emergency transport at the community level, as well as the provision of water and electricity in the facilities.

Water and electricity are crucial for the effective operation of facilities. Water helps maintain hygiene and sanitation in facilities, while electricity facilitates the sterilisation of equipment as well as storage of drugs, vaccines and associated adjuvants [37, 38]. The World Health Organization (WHO) considers WASH (Water, Sanitation and Hygiene) services in facilities as very necessary for the attainment of the SDGs, especially those relating to maternal and child health [39]. This explains the inclusion of WASH services in the framework for quality of care for maternal and child health.

#### Lesson number four: lack of essential inputs and infrastructure impeded quality care

Quality of care is compromised by the lack of essential inputs and infrastructure in facilities. Poor quality of care not only discourages women from service usage, but does not permit the achievement of good health outcomes. For instance, implementing fee free policies may lead to an increase in the use of services but maternal deaths may not reduce proportionately if the quality of care is poor [10]. All pregnant women need to be provided with quality care at pregnancy, labour, birth and beyond [40, 41]. The WHO’s framework for quality of care stipulates the need for continuous assessment, improvement and monitoring within the health system. It is crucial to ensure the availability of the necessary inputs for quality care provision [41], including an adequate workforce and skilled, regulated and educated midwives [42].

In conclusion, lessons from our study included the persistence of OOP payments, a vulnerable health system, poorly resourced lower level facilities and low quality of care due to the lack of essential inputs and infrastructure. These negatively affect the drive towards reducing maternal and child deaths and the attainment of UHC. It is suggested that the Government of Ghana, the NHIS and other stakeholders improve the provision of resources to facilities, especially lower level ones.

## Limitations of the study

The study has its limitations. Firstly, the estimated levels of OOP payments might be underestimated, as productivity losses for women and their caregivers were not determined. Secondly, recall bias on the part of the women cannot be ruled out since the interviews and discussions were held after women had given birth.

## Abbreviations

CHPS: community-based health planning and services
MDGs: Millennium Development Goals
NHIS: National Health Insurance Scheme
OOP: out of pocket
UHC: universal health coverage
SDGs: Sustainable Development Goals
WASH: Water, Sanitation and Hygiene
WHO: World Health Organization
